# Fitness Cost of Resistance to Bt Cotton Linked with Increased Gossypol Content in Pink Bollworm Larvae

**DOI:** 10.1371/journal.pone.0021863

**Published:** 2011-06-30

**Authors:** Jennifer L. Williams, Christa Ellers-Kirk, Robert G. Orth, Aaron J. Gassmann, Graham Head, Bruce E. Tabashnik, Yves Carrière

**Affiliations:** 1 Department of Entomology, University of Arizona, Tucson, Arizona, United States of America; 2 Monsanto LLC, St. Louis, Missouri, United States of America; 3 Department of Entomology, Iowa State University, Ames, Iowa, United States of America; Ghent University, Belgium

## Abstract

Fitness costs of resistance to *Bacillus thuringiensis* (Bt) crops occur in the absence of Bt toxins, when individuals with resistance alleles are less fit than individuals without resistance alleles. As costs of Bt resistance are common, refuges of non-Bt host plants can delay resistance not only by providing susceptible individuals to mate with resistant individuals, but also by selecting against resistance. Because costs typically vary across host plants, refuges with host plants that magnify costs or make them less recessive could enhance resistance management. Limited understanding of the physiological mechanisms causing fitness costs, however, hampers attempts to increase costs. In several major cotton pests including pink bollworm (*Pectinophora gossypiella*), resistance to Cry1Ac cotton is associated with mutations altering cadherin proteins that bind this toxin in susceptible larvae. Here we report that the concentration of gossypol, a cotton defensive chemical, was higher in pink bollworm larvae with cadherin resistance alleles than in larvae lacking such alleles. Adding gossypol to the larval diet decreased larval weight and survival, and increased the fitness cost affecting larval growth, but not survival. Across cadherin genotypes, the cost affecting larval growth increased as the gossypol concentration of larvae increased. These results suggest that increased accumulation of plant defensive chemicals may contribute to fitness costs associated with resistance to Bt toxins.

## Introduction

Corn and cotton engineered to produce insecticidal proteins from *Bacillus thuringiensis* (Bt) can increase agricultural profitability while reducing reliance on insecticide sprays [1], [2]. However, field-evolved resistance to toxins in Bt crops, which has been reported in several species of major insect pests, threatens these benefits [3]–[7]. Analysis of global monitoring data suggests that host plants that do not make Bt toxins and grow near Bt crops can reduce the risk of resistance [2]–[4]. Such non-Bt plant “refuges” can provide many susceptible individuals to mate with the rare resistant individuals surviving on Bt crops, yielding hybrid offspring. Refuges are expected to delay resistance most effectively when resistance is inherited as a recessive trait, so the hybrid offspring are killed on Bt crops [4], [8], [9].

Fitness costs of resistance occur in the absence of Bt toxins through pleiotropic effects that reduce fitness of individuals carrying resistance alleles relative to susceptible individuals that lack such alleles [10]. Because costs of Bt resistance are common, refuges not only delay resistance by providing susceptible individuals to mate with resistant individuals, but also by selecting against resistance alleles [10]–[14]. Costs are modulated by variation in environmental conditions, including host plants, competition, overwintering, and natural enemies [10]. Accordingly, refuges that magnify costs or make them less recessive could enhance resistance management [9], [10], [14]. Although dozens of studies have documented fitness costs of Bt resistance that affect many life history traits, little is known about the physiological mechanisms that cause such costs [10]. In particular, the mechanisms underlying variation in costs among host plants are not well understood, which hampers attempts to identify or create refuge plants that magnify costs.

Knowledge of the molecular and genetic basis of resistance to Bt toxins is essential for understanding what causes fitness costs and why such costs vary among host plants. In pink bollworm, *Pectinophora gossypiella*, and two other major lepidopteran pests of cotton, mutations in genes encoding cadherin proteins that bind Bt toxins are associated with resistance to Bt toxins [15]–[17]. In pink bollworm, three mutant alleles (*r1*, *r2,* and *r3*) linked with resistance to Bt toxin Cry1Ac encode incomplete versions of a cadherin protein that binds Cry1Ac in susceptible larvae [16], [18]. The normal role of Bt toxin-binding cadherin proteins, which occur in the larval midgut, remains unclear. They may affect the morphology of microvilli on the apical surface of midgut cells through several processes, including enhancement of cell adhesion and guidance of cell differentiation [19]–[22]. If cadherin mutations conferring resistance to Bt toxins interfere with midgut functional or structural integrity, such mutations could cause fitness costs by increasing the absorption and concentration of plant defensive chemicals in insect larvae.

Here we tested the hypothesis that fitness costs of resistance to Bt cotton in pink bollworm are associated with increased concentration in larvae of a plant defensive chemical, gossypol. Gossypol is a polyphenolic aldehyde from cotton (*Gossypium* spp.) that is toxic to many insects and plant pathogens [23]–[25]. Although the mechanisms responsible for gossypol toxicity to insects remain unknown, gossypol in the hemolymph may permeate cells and cause toxic effects on many life history traits. We reported previously that adding gossypol to the larval diet of pink bollworm reduced performance and increased fitness costs [23], but we did not measure the concentration of gossypol in larvae or identify their cadherin genotype. The results reported here confirm that gossypol reduces larval performance. We discovered that gossypol concentration was higher in larvae with cadherin resistance alleles than in larvae without such alleles. The results also show that across larval cadherin genotypes, increased gossypol concentration was associated with higher fitness costs.

## Results

### Effects of Cadherin Genotype on Larval Gossypol Concentration

As expected, gossypol was not detected in any larvae from control diet without gossypol, either from the related susceptible strain (MOV97H1-S) and resistant strain (MOV97-H1R) in experiment one, or from the hybrid strain (MOV97-H3) in experiment two (Table 1, n = 10). In contrast, across the two experiments, gossypol was detected in 93% of larvae from gossypol-treated diet (n = 175).

**Table 1:** Mean larval weight (mg) and gossypol concentration (µg/g dry weight) on gossypol and control diet in cadherin genotypes from experiment one (MOV97-H1S and MOV97-H1R) and experiment two (MOV97-H3).

Diet	Genotype	Larval weight^1,3^	n^2^	Gossypol concentration^1,3^	n^2^
Experiment 1: MOV97-H1S and MOV97-H1R					
Control	*ss*	30.5 (0.3)	311	0	1
	*r3r3*	28.6 (0.5)	113	0	1
	*r1r3*	27.9 (0.6)	100	0	1
	*r1r1*	28.6 (0.5)	65	0	1
	*rr (averaged)*	28.3 (0.4)	3	0	3
Gossypol	*ss*	28.8 (0.3)	232	0.56 (0.08)	25
	*r3r3*	26.0 (0.5)	85	1.06 (0.14)	25
	*r1r3*	25.1 (0.6)	80	2.09 (0.32)	23
	*r1r1*	23.7 (0.7)	50	4.87 (1.15)	12
	*rr (averaged)*	24.9 (0.6)	3	2.67 (1.14)	3
Experiment 2: MOV97-H3					
Control	*ss*	29.9 (0.7)	61	0	1
	*r3s*	27.3 (0.6)	86	0	1
	*r1s*	30.7 (0.8)	39	0	1
	*r3r3*	28.1 (1.0)	33	0	1
	*r1r3*	30.3 (0.9)	32	0	1
	*r1r1*	28.7 (1.0)	24	0	1
	*rs (averaged)*	29.0 (0.7)	2	0	2
	*rr (averaged)*	29.0 (0.9)	3	0	3
Gossypol	*ss*	28.8 (0.8)	56	0.41 (0.08)	22
	*r3s*	25.7 (0.7)	77	1.43 (0.08)	31
	*r1s*	24.7 (1.1)	33	2.17 (0.16)	14
	*r3r3*	25.4 (1.2)	28	2.06 (0.24)	12
	*r1r3*	31.0 (1.5)	21	3.52 (0.76)	9
	*r1r1*	20.2 (5.8)	4	11.21 (3.80)	2
	*rs (averaged)*	25.2 (0.9)	2	1.80 (0.37)	2
	*rr (averaged)*	25.5 (2.8)	3	5.60 (2.84)	3

1 For each combination of genotype and diet, standard errors are reported in parentheses.

2 For each combination of genotype and diet, number of larvae (n) are reported.

3 The means for *rr* are the average across *r3r3*, *r1r3*, and *r1r1*; for *rs* these are the average across *r3s* and *r1s*.

**Figure 1:**
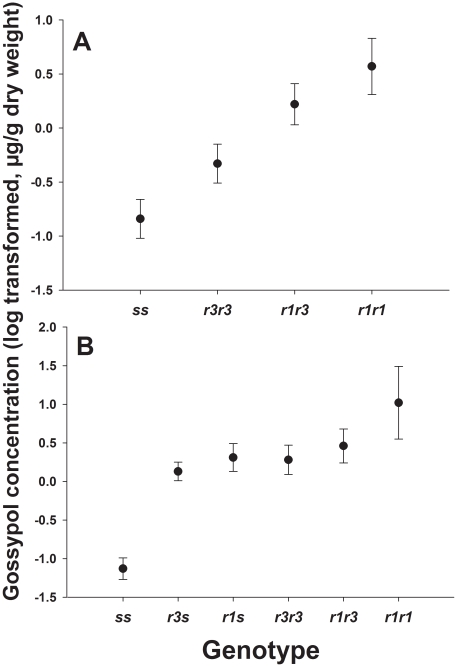
Gossypol concentration in pink bollworm cadherin genotypes. Mean gossypol concentration (±SE, log [x+0.001] transformed) in larvae from (A) MOV97-H1S and MOV97-H1R and (B) MOV97-H3 fed on gossypol diet.

Across both experiments, the proportion of larvae from gossypol diet in which we detected no gossypol was significantly higher for susceptible (*ss*) larvae (10/47 = 0.21) than for larvae with either one *r* allele (*rs*) or two *r* alleles (*rr*) (3/128 = 0.02) (Fisher's exact test, P<0.0001). For larvae fed gossypol diet in both experiments, gossypol concentration was higher in *rs* and *rr* larvae than in *ss* larvae (Fig. 1 and Table 1). In experiment one, gossypol concentration was 4.8 times higher in *rr* (2.67 µg/g) than *ss* (0.56 µg/g) (t = 4.58, df = 81, P<0.0001). In experiment two, relative to the gossypol content of *ss* (0.41 µg/g), gossypol content was 13.6 times higher in *rr* (5.60 µg/g) and 4.4 times higher in *rs* (1.80 µg/g). Gossypol content was significantly higher in *rr* than *ss* (t = 6.35, df = 84, one-sided P<0.0001) and in *rs* than *ss* (t = 7.55, df = 84, one-sided P<0.0001), but did not differ significantly between *rs* and *rr* (t = 1.67, df = 84, P = 0.098). Because gossypol content of *rs* differed from *ss* but not *rr*, inheritance of this trait was not recessive. In both experiments, gossypol concentration differed between each *rs* or *rr* genotypes and *ss* (Fig. 1A, one-sided P values<0.024; Fig. 1B, one-sided P values<0.0001).

### Effects of Gossypol and Cadherin Genotype on Larval Weight

**Figure 2:**
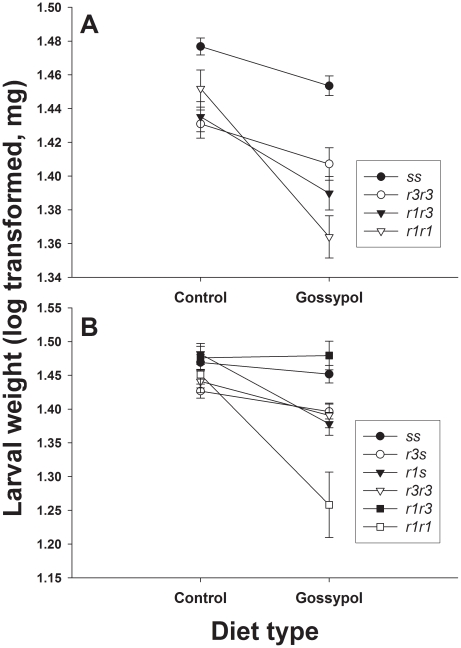
Weight of pink bollworm cadherin genotypes on control and gossypol diet. Mean weight (±SE, log transformed) of larvae from (A) MOV97-H1S and MOV97-H1R and (B) MOV97-H3.

In both experiments, larval weight was lower on gossypol diet than on control diet. Also, in both experiments, the reduction in larval weight on gossypol diet relative to control diet was greater for *rs* and *rr* larvae than for *ss* larvae. In experiment one on control diet, weight was lower in *rr* (28.3 mg) than *ss* (30.5 mg) (t = 5.04, df = 1028, P<0.0001), showing a fitness cost without gossypol. On gossypol diet, weight was also lower in *rr* (24.9 mg) than *ss* (28.8 mg) (t = 7.81, df = 1028, one-sided P<0.0001). The coefficient of the linear contrast with associated 95% confidence interval was −0.037 (−0.49, −0.25) on control diet and −0.67 (−0.081, −0.053) on gossypol diet. As each coefficient lies outside the confidence interval for the other, costs were significantly higher on gossypol than control diet (P<0.05). Larval weight was generally lower on gossypol diet than control diet (Fig 2A, t = −6.17, one-sided P<0.0001). Relative to *ss*, weight differed more between gossypol diet and control diet in *r1r1* (t = −3.52, one-sided P<0.00025), but not in the other genotypes (one-sided P values>0.08) (Fig. 1A).

In experiment two on control diet, weight of *rr* (29.0 mg) did not differ significantly from *ss* (29.9 mg) (t = 0.80, df = 482, P = 0.42), nor did weight of *rs* (29.0 mg) differ from *ss* (t = 0.96, df = 482, P = 0.34). Thus, no cost affecting weight was seen on control diet in this experiment. On gossypol diet, however, weight was lower for *rr* (25.5 mg) than *ss* (28.8 mg) (t = 3.33, df = 482, one-sided P = 0.00045), which indicates a cost. Furthermore, weight was lower for *rs* (25.2 mg) than *ss* (t = 3.93, df = 482, one-sided P<0.0001), but weight of *rs* and *rr* did not differ significantly (t = 0.53, df = 482, P = 0.59). Thus, gossypol induced a non-recessive cost affecting larval weight. Larval weight was lower on gossypol diet than control diet (Fig. 2B, t = −5.11, one-sided P<0.0001). Relative to *ss*, weight differed more between gossypol diet and control diet in *r1r1* (t = −3.18, one-sided P = 0.008) and *r1s* (t = −2.96, one-sided P = 0.002), but not in the other genotypes (one-sided P values>0.14) (Fig. 1B).

**Figure 3:**
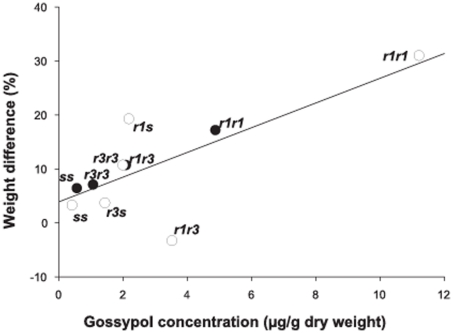
Association between gossypol concentration and difference in weight on gossypol diet relative to control diet. Closed circles show data from MOV97-H1S and MOV97-H1R; open circles show data from MOV97-H3.

Pooling *rr* and *rs* genotypes across experiments, the mean cost (±SE) affecting weight was significantly different from zero both on control diet (5.3±1.7%; t = 3.04, df = 7, P = 0.019) and gossypol diet (12.9±3.7%; t = 3.52, df = 7, one-sided P = 0.0048). The increase in cost from control to gossypol diet was statistically significant (paired t-test, t = 1.98, df = 7, one-sided P = 0.044). Across the six larval genotypes tested in experiments one and two, a positive association occurred between gossypol content and the decrease in larval weight on gossypol diet compared to control diet (Fig. 3, t = 3.23, df = 8, one-sided P = 0.0060). The association between gossypol content and decreased weight was also significant when data were analyzed separately for experiment one (P = 0.0015) and two (P = 0.046). This suggests that increased gossypol concentration in larvae with one or two *r* alleles compared to *ss* increased the cost affecting larval growth.

### Effects of Gossypol and Cadherin Genotype on Survival

**Figure 4:**
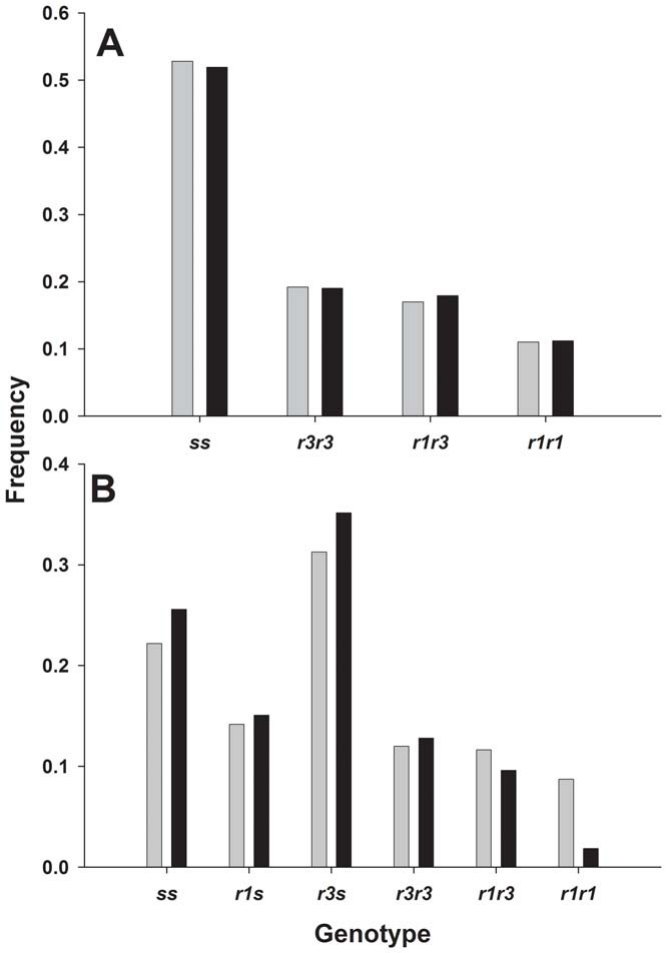
Frequency of cadherin genotypes on control diet (grey bars) and gossypol diet (black bars). (A) MOV97-H1S and MOV97-H1R and (B) MOV97-H3. For each type of diet, frequency for each genotype was calculated as the number of survivors of that genotype divided by total number of survivors.

Similar to the results with larval weight, larval survival was lower on gossypol diet than on control diet in both experiments. In experiment one, survival was lower on gossypol diet (27.9%) than on control diet (36.8%) (one-sided P<0.0001). On control diet, survival of *ss* (38.9%) did not differ significantly from survival of *rr* (34.8%) (Table 1, P = 0.10). On gossypol diet, survival of *ss* (29.0%) also did not differ significantly from survival of *rr* (26.9%) (one-sided P = 0.18). Thus, gossypol did not increase the survival cost. The difference in genotype frequency on gossypol diet relative to control diet did not differ significantly between *ss* and *r1r1*, *r1r3*, or *r3r3* (Fig. 4 A, one-sided P values>0.34), which suggests that gossypol did not reduce survival of any of the *rr* genotypes relative to *ss*.

In experiment two, survival was lower on gossypol diet (27.4%) than on control diet (34.4%) (one-sided P = 0.0014). We could not test directly for a fitness cost in experiment two (see Methods), but we could determine if the frequency of *rs* and *rr* relative to *ss* was reduced more on gossypol diet than on control diet. Compared to control diet, the frequency of *rr* (vs. *ss*) was marginally reduced on gossypol diet (one-sided P = 0.057), although the frequency of *rs* remained similar on control and gossypol diet (one-sided P = 0.45). The reduction in frequency on gossypol diet relative to control diet was significantly greater in *r1r1* than *ss* (Fig. 4 B, χ^2^ = 11.73, one-sided P = 0.0003), but not in other genotypes with *r* alleles (all one-sided P values>0.15). This suggests that gossypol reduced survival of *r1r1* relative to *ss.*

## Discussion

We focused here on the effects of the cotton defensive compound gossypol on fitness costs by feeding Bt-resistant and susceptible pink bollworm larvae on diet without Bt toxin that either contained or lacked gossypol. We found that when pink bollworm larvae ate diet containing gossypol, the concentration of gossypol was higher in larvae with cadherin alleles conferring resistance to Cry1Ac than in susceptible larvae lacking such alleles (Fig. 1). In both experiments, costs affecting larval growth rate, as indicated by weight of 14-day-old larvae, were magnified on gossypol diet (Table 1; Fig. 2). In experiment two, larval gossypol concentration was non-recessive; it was higher in *rs* larvae than in *ss* larvae (Fig. 1). In addition, in this experiment, costs affecting growth were absent on control diet but dominant on gossypol diet (Table 1). These results show that higher larval gossypol content was associated with a non-recessive cost affecting larval growth. This is potentially important because non-recessive costs select more strongly against resistance than recessive costs [9]–[14].

When results from both experiments were considered, costs affecting growth were significantly higher on gossypol diet (12.9%) than on control diet (5.3%). Furthermore, a significant, positive association across genotypes occurred between larval gossypol concentration and the decrease in weight on gossypol diet relative to control diet (Fig. 3). These results show that cadherin mutations conferring resistance to Cry1Ac cotton in pink bollworm were associated with higher larval gossypol concentrations and a higher fitness cost affecting larval growth.

The strains MOV97-H1S and MOV97-H1R and MOV97-H3 had a common origin and contained similar *r* and *s* alleles, but differed in their rearing history. Costs reducing larval growth on control diet were present in MOV97-H1S and MOV97-H1R but not in MOV97-H3 (Fig. 2). Also, relative to *ss*, the frequency of *r1r1* decreased more on gossypol than control diet in MOV97-H3 but not in MOV97-H1S and MOV97-H1R (Fig. 4). Such differences in the response of the same genotypes from different strains indicate that variation in genetic background affected costs. The *r1* allele has a deletion of 24 base pairs resulting in two amino acid substitutions and the elimination of eight amino acids. The *r3* allele has a deletion of 126 base pairs resulting in the omission of 42 amino acids [16]. Across all of the strains studied, we found that effects of gossypol in diet on larval gossypol concentration and growth were greater for *r1* than *r3* (Fig. 1, 2 and 3).

The results reported here confirm results from a previous study of fitness costs in three strains of pink bollworm derived from MOV97: a susceptible strain, a Cry1Ac-resistant strain, and their F1 progeny [23]. In the previous study, larval gossypol concentration was not measured and cadherin genotypes were not identified. However, similar to the results reported here, gossypol in diet significantly increased the cost affecting larval developmental time, but did not increase the cost affecting survival [23].

In general, costs of Bt resistance are significantly higher on plants than on diet [10]. Compared with the mean costs from many studies [10], the cost affecting growth on control diet seen here (5.3%) is virtually identical to the mean cost affecting development time on diet (5.0%), while the cost affecting growth on gossypol diet seen here (12.9%) is somewhat higher than the mean cost affecting developmental time on plants (8.9%).

Although it was hypothesized that fitness costs are higher on host plants with low suitability [26], [27], available data show no consistent relationship between costs of Bt resistance and host plant suitability [10]. In *Trichoplusia ni*, costs were negatively associated with host plant suitability, supporting this hypothesis [26]. In *Plutella xylostella*, however, the cost affecting development time was greater on the least suitable host plant, but the survival cost was higher on the most suitable host in one of the two *P. xylostella* populations investigated [27]. Furthermore in *H. armigera*, cotton, pigeon pea, and sorghum were equally suitable to *ss* individuals, but costs were larger or less recessive on sorghum and cotton than pigeon pea [28]. In *P. gossypiella*, survival was significantly greater on the cotton cultivar DP50 with high gossypol content than on TX53 with low gossypol content, but the cost affecting survival was recessive and of similar magnitude on both cultivars [29]. High content of defensive chemicals other than gossypol could have suppressed survival on TX53, however, and these chemicals could have compensated for effects of high gossypol content in DP50.

In the studies cited above, among-host variation in nutrient availability and defensive chemicals was not well characterized, and the molecular basis of resistance was known only in pink bollworm. Resistance to Bt toxins is associated with mutations affecting ABC transporter, aminopeptidase-N, and cadherin proteins that act as toxin receptors in the midgut, as well as with proteases that convert Bt protoxins to activated toxins [30], [31]. Mutations affecting aminopeptidase-N and proteases, which are two major digestive proteases, could impair protein digestion [10]. Mutations altering ABC transporters could contribute in reducing export of toxic compounds by midgut epithelial cells to the gut lumen [31], while cadherin mutations could contribute in increasing concentrations of plant defensive chemicals in larvae, as suggested by the results here. Accordingly, low availability of nutrients could be the main factor increasing costs in insects with aminopeptidase-N- and protease-mediated resistance, as it would be harder to compensate for digestive deficiencies when nutrient availability is low rather than high. In contrast, high concentration of defensive chemicals could be the most important reason for an increase in costs in insects with ABC transporter- and cadherin-mediated resistance. Therefore, future studies on variation in costs across host plants may benefit from a more mechanistic approach.

Functional studies of larvae with various cadherin genotypes are needed to determine the effects of cadherin mutations on midgut structure and permeability to plant defensive chemicals. To better understand how host plants affect costs, it will also be necessary to evaluate the relationships among defensive compounds, nutrient availability, and fitness when Bt-susceptible and -resistant insects develop on their natural host plants. Cotton was recently genetically engineered to produce plants with little gossypol in seeds but normal levels in stems and leaves [32]. As pink bollworm larvae primarily feed on seeds, these transgenic cultivars provide an ideal system to further assess the interaction between cadherin-based resistance to Bt cotton, absorption of defensive compounds and expression of fitness costs.

## Materials and Methods

### Insect Strains

We used three pink bollworm strains derived from strain MOV97, which was established from a single collection in Mohave Valley, Arizona in 1997 [33]. At the locus encoding a cadherin protein that binds Bt toxin Cry1Ac, MOV97 had two alleles (*r1* and *r3*) that confer recessive resistance to Bt toxin Cry1Ac and transgenic cotton that produces Cry1Ac [16], [34]. At this locus, MOV97 also had *s* alleles that confer susceptibility to Cry1Ac [16], [34]. We conducted two independent experiments: In experiment one, we used two strains that had a similar genetic background, yet one was resistant (MOV97-H1R) and contained only the *r1* and *r3* alleles, while the other was susceptible (MOV97-H1S) and contained only *s* alleles. In experiment two, we used a hybrid strain (MOV97-H3) in which the *r1*, *r3* and *s* alleles segregated at the cadherin locus. We refer to individuals with any two *r* alleles (*r1r1*, *r3r3*, or *r1r3*) as *rr*, and to individuals with one *r* allele (*r1s* and *r3s*) as *rs*.

MOV97-H1R, MOV97-H1S and MOV97-H3 were derived from the hybrid strain MOV97-H1. MOV97-H1 was produced by crossing a resistant strain (MOV97-R) and a susceptible strain (MOV97-S) that had been derived from MOV97 [34]. MOV97-H1R was produced by exposing larvae of the F18 generation of MOV97-H1 to a diagnostic concentration of Cry1Ac in synthetic diet (10 µg toxin per ml diet) that allows survival of only *rr* individuals [35], [36]. MOV97-H1S was initiated by screening mated pairs of MOV97-H1 (F17) with PCR to find pairs of *ss* individuals [36]. Ten mated pairs with either *rr* or *ss* individuals were caged individually to initiate MOV97-H1R and MOV97-H1S. From each of the 10 mated pairs, 12 pupae were collected (120 per strain). The adult population size was 378 per strain in the F2 generation and 1200 in the following generations [36]. MOV97-H3 was created by crossing insects from MOV97-H1 (F22), which were predominantly *ss* and *rs*, with a subset of insects from MOV97-H1 (F21) that had been selected with the diagnostic concentration of Cry1Ac and thus were resistant [34]. Adult population size in MOV97-H1 and MOV97-H3 was>1000 in every generation.

Experiment one was conducted in September 2005 with F4 larvae of MOV97-H1S and MOV97-H1R. Experiment two was done in November 2006 with F5 larvae of MOV97-H3. The parental strain MOV97-H1 had been reared for 17–18 generations on non-Bt diet before MOV97-H1S and MOV97-H1R were created, and 21–22 generations before MOV97-H3 was created. Also, before experiments were done, each of these three strains had been reared for either four or five generations on non-Bt diet. This rearing reduced linkage disequilibrium between cadherin alleles and other alleles that may affect fitness [37].

### Insect Diet

To investigate the effects of gossypol, newly hatched larvae were placed on wheat germ diet [38] with gossypol (gossypol diet) or without gossypol (control diet). For gossypol diet, we added gossypol (95% in acetic acid crystal, Sigma-Aldrich) in 1 ml of hexane to achieve a concentration of 0.1% fresh weight (0.1 g gossypol/100g fresh weight) [29]. This is equivalent to 0.6% dry weight, a concentration within the range that occurs naturally in seeds of *Gossypium* spp. [29], [39], [40]. For control diet, we added the same amount of hexane without gossypol. We also made red diet without gossypol by adding red calco dye (Bio-Serv, Frenchtown, NJ, USA) to control diet at a concentration of 0.010% [41]. We used the red diet to monitor voiding of gossypol diet from the larval gut (see below).

### Effect of Gossypol Diet on Costs

We randomly assigned neonate larvae of the susceptible strain (MOV97-H1S, n = 800), the resistant strain (MOV97-H1R, n = 800) and the hybrid strain (MOV97-H3, n = 400) to diet with or without gossypol. We reared larvae in trays with 16 wells (15 mm deep, 3 ml in volume). We put 1.5 g of diet and one neonate in each well. Each tray was sealed with a transparent cover with holes for ventilation. Trays containing one of the two diet types were randomly allocated to shelves in a growth chamber maintained at 27±2°C with ambient relative humidity and a photoperiod of 14∶10 (L∶D) h.

After feeding for 14 days, survivors from both diet types were placed on red diet for 1.5 h to void their gut of the undyed diet. Preliminary work showed that 1.5 h was sufficient to void the gut of diet. After 1.5 h on red diet, each larva was scored for mortality and weighed. The top half of the head capsule of each larva was excised with a razor blade such that no hemolymph was lost, and preserved in 100% EtOH for subsequent genotyping. Larvae were then freeze dried with liquid nitrogen, placed in glass extraction vials (KIMAX brand sample vials, borosilicate glass, with PTFE-lined screw cap 4 ml), and stored at −80 C until gossypol was extracted and quantified.

### Insect Genotyping

We extracted DNA from individual larvae using the protocol from Morin et al. [16] with slight modifications [42]. We determined larval cadherin genotype using PCR with primer sets that selectively amplify each of the four types of cadherin alleles (*r1*, *r2*, *r3,* and *s*) [43]. As expected, no *r2* alleles were found in any of the larvae genotyped.

### Gossypol Analyses

We measured gossypol concentration in individual larvae by creating an aniline Schiff's base and quantifying the resulting dianilino-gossypol complex with high pressure chromatography coupled with a triple quadrupole mass spectrometer [44]. We used an external calibration curve and an internal standard. We assumed extraction efficiency was 100%. The lowest detectable concentration was 0.025 µg/g. Values below this limit are reported as zero.

In experiment one, a total of 89 larvae from MOV97-H1S and MOV97-H1R were sent to Monsanto labs (Creve Coeur, MO) for analysis. These comprised a random sample of 25 MOV97-H1S larvae fed on gossypol diet, a random sample of 60 MOV97-H1R larvae fed on gossypol diet, one MOV97-H1S larva fed on control diet selected at random from *ss* larvae previously identified with PCR, and one of each of *r1r1*, *r1r3* and *r3r3* from MOV97-H1R fed on control diet and selected randomly from larvae previously identified with PCR. In the second experiment, a total of 96 MOV97-H3 larvae were sent to Monsanto labs. These comprised 90 randomly selected larvae that had fed on gossypol diet, and one larva from each genotype (i.e., *ss*, *r1s*, *r3s*, *r1r1*, *r1r3* and *r3r3*) fed on control diet and selected randomly from larvae identified previously with PCR. Larvae were shipped overnight on dry ice and remained frozen for the duration of the shipment. Gossypol analyses were conducted without knowledge of genotype or experimental diet, as the shipped samples were identified only by a number.

### Statistical Analyses

We used statistical analyses to test our main hypotheses: 1) for larvae fed gossypol diet, gossypol concentration is lower in *ss* larvae than in *rr* and *rs* larvae; 2) gossypol reduces performance of all larvae as indicated by lower larval weight or lower survival; 3) the extent of reduction in larval performance on gossypol diet relative to control diet is greater for *rr* and *rs* larvae than for *ss* larvae. As each of these *a priori* hypotheses specifies the direction of the difference between groups, we report one-tailed P values for all tests of these hypotheses.

In each experiment, one-way ANOVA followed by linear combinations of means (hereafter linear contrasts) were used to assess whether gossypol concentration (µg/g dry weight, transformed log x+0.001) differed among the *ss*, *rs*, and *rr* genotypes. Multiple regression with indicator variables for each genotype with *r* allele (s) was further used to compare gossypol concentration between *r1r1*, *r1r3*, *r1r1* and *ss* in experiment one and between *r1s*, *r3s*, *r1r1*, *r1r3*, *r3r3* and *ss* in experiment 2.

In each experiment, two-way ANOVA followed by linear contrasts was used to evaluate whether larval weight (fresh weight in mg, log transformed) differed among the *ss*, *rs*, and *rr* genotypes. In experiment one, costs on control and gossypol diet were compared by assessing whether each contrast coefficient (i.e., *ss* vs. *rr*) lied outside the 95% confidence interval for the other [45]. Multiple regression with indicator variables for each genotypes with *r* allele (s) and gossypol diet was further used to assess the effects of diet (gossypol versus control diet), genotype, and the interaction between these factors on larval weight (fresh weight in mg, log transformed) in each experiment. A significant negative coefficient associated with the interaction between a particular genotype and diet indicated that larval weight of this genotype was reduced more (relative to *ss*) on diet with gossypol than on control diet [45].

We measured costs affecting larval weight of a genotype with *r* alleles on a particular diet as: cost in % = 100%× ([mean weight (genotype) – mean weight *ss*] / mean weight *ss*). As costs did not differ between experiments on either diet (2-sample t-test, P values>0.15), we pooled genotypes from both experiment and used a one-sample t-test to assess whether costs were significantly different from zero on control and gossypol diet. We also used a paired t-test to evaluate whether costs were significantly greater on gossypol than control diet.

For each genotype, we calculated the percentage reduction in weight from control to gossypol diet as: 100%× ([mean weight on control diet – mean weight on gossypol diet]/mean weight on control diet). We used a covariance analysis to test for effects of experiment, gossypol concentration in a genotype, and the interaction between these factors on the change in weight from control to gossypol diet. Because the effects of experiment (P = 0.61) and interaction (P = 0.93) were not significant, we pooled data and used linear regression to assess the association between gossypol content and change in weight from control to gossypol diet. We also used linear regression to evaluate the association between gossypol content and change in weight from control to gossypol diet separately for experiment one and two.

In both experiments, we used a Fisher's exact test to evaluate whether the proportion of larval survival differed between gossypol and control diet. To test for fitness costs affecting survival in experiment one, we pooled the *rr* genotypes and used a Fisher's exact test to determine if the proportion of survival of *ss* and *rr* differed on gossypol or control diet. We could not measure survival of genotypes in experiment two because we did not know the initial number of individuals of each genotype, only the number of survivors of each genotype. Nevertheless, in both experiments, we assessed whether the frequency of *rr* or *rs* decline more from control to gossypol diet than the frequency of *ss*. In experiment two, we used a Fisher's exact test to compare the frequency of *rr* (three genotypes pooled) and *rs* (two genotypes pooled) to the frequency of *ss* on each diet (gossypol and control). In both experiments, we also used log-linear regression to assess whether frequency decreased more on gossypol than control diet in each genotype with *r* allele (s) than is *ss*. Explanatory variables in the regression model were indicator variables for diet, genotype, and the interaction between these factors, while the response variable was the number of individuals of each genotype surviving on the diet types. A significant negative coefficient associated with the interaction between a particular genotype and diet indicated that frequency of this genotype was reduced more (relative to *ss*) on diet with gossypol than on control diet [45]. Statistical analyses were performed in JMP [46].
